# Genome sequencing reveals a highly divergent Biotype II African swine fever virus in Nigeria

**DOI:** 10.21203/rs.3.rs-9912866/v1

**Published:** 2026-06-22

**Authors:** Alhaji S. Olono, Olusola A. Ogunsanya, Femi M. Saibu, Vivian K. O’Donnell, Steven M. Lakin, Edyth Parker, Roger W. Barette, Lizhe Xu, Oluwatobi A. Adedokun, John Fadele, John O. Abiola, Eugenie Y. Tchokote, Danny Park, Gerald Mboowa, Christopher H. Tomkins-Tinch, Faburay Bonto, Corrie Brown, Christian T. Happi, Anise N. Happi

**Affiliations:** Redeemer’s University Akoda-Ede; Redeemer’s University Akoda-Ede; Redeemer’s University Akoda-Ede; United States Department of Agriculture; United States Department of Agriculture; Redeemer’s University Akoda-Ede; United States Department of Agriculture; United States Department of Agriculture; Redeemer’s University Akoda-Ede; Redeemer’s University Akoda-Ede, Osun State, Nigeria.; University of Ibadan; Michael Okpara University of Agriculture; Broad Institute; Broad Institute; Broad Institute; United States Department of Agriculture; LifeStock International; Redeemer’s University Akoda-Ede; Redeemer’s University Akoda-Ede

**Keywords:** whole genome, sequencing, characterization, African swine fever virus, outbreak, phylogenetics, Nigeria

## Abstract

African swine fever is a viral disease with major socioeconomic consequences in countries including Nigeria, where pig farming sustains many livelihoods. Despite the critical role of genomic data in outbreak control, Nigeria has remained underrepresented in global African swine fever virus (ASFV) genomic datasets. Here, we report the first in-country sequencing and genomic analysis of ASFV from recent outbreaks in Nigeria, representing the largest dataset of ASFV genomes generated in Nigeria and Africa to date. Using Oxford Nanopore and Illumina Technology platforms, we assembled 27 whole-genome sequences from field samples collected in Ogun, Osun, and Oyo States. Comparative analyses revealed nearly 100 single-nucleotide polymorphisms (SNPs) in coding regions and more than 250 across coding and non-coding regions, alongside multiple insertions and deletions relative to the Georgia 2007 ASFV reference genome. Notably, we report novel large deletions in variable regions, which may affect genes linked to virulence and host adaptation. Phylogenetic analysis revealed that the Nigerian ASFV genomes form a distinct clade within biotype II, which also diverges considerably from the genome backbone currently utilized for vaccine designs, highlighting the impact of viral divergence on current control measures.

## Introduction

The livestock industry is currently facing a huge panzootic-scale threat: African swine fever (ASF). This highly contagious viral hemorrhagic disease affects both domestic and wild suids, with mortality rates in domestic pigs reaching up to 100%^1^. Currently, ASF is present across multiple countries in Asia, Africa, the Caribbean (knocking at the doors of both North and South America), Europe, and the Pacific^2,3^. The scale of recent outbreaks has been devastating, with the 2018 epidemic in China leading to the loss of an estimated 225 million pigs^4^. Anecdotal evidence suggests that the 2020 epidemic in Nigeria alone resulted in the death of nearly one million pigs, affecting the livelihood of close to 3,000 farmers^5–7^. The spread of ASF has profound economic and food security implications, disrupting both local and global pig production and pork trade^8–10^. Beyond economic losses, the disease threatens biodiversity by reducing wild boar and domestic pig populations^11^.

Current preventive and control measures rely on strict biosecurity, early pathogen detection, culling of infected herds, and the imposition of restriction zones and movement controls on pigs and pig products^12,13^. While these approaches are necessary, they are also associated with substantial socio-economic costs, especially in low and middle-income countries such as Nigeria^8,10^. The vast panzootic footprint of ASF, combined with the burden of current control strategies, highlights the urgent need for a concerted global response towards eliminating or reducing the disease. Achieving success in such efforts will depend largely on a comprehensive understanding of the causative African Swine Fever Virus (ASFV), particularly at the genetic and genomic levels across affected regions.

Africa plays a pivotal role in this context. The continent is not only the endemic home of ASFV but also harbors all the 24 known viral genotypes as described by previous p72 gene sequencing and classification approaches^14–16^. Gene-based genotyping has been a very important part of tracking and understanding ASF epidemiology globally^17,18^. However, due to the methodological issues observed with this genotyping approach, efforts have been made in recent times to recharacterize the pathogen both from an updated p72 gene and whole genome-based classification angles. Based on these, ASFV can now be divided into six (instead of 24) and seven distinct p72 genotypes and whole genome-based biotypes, respectively^19,20^. The updated p72 classification, although arriving at a smaller number of genotypes, still recognizes that ASFV is a diverse pathogen^20^. It emphasizes that the p72 (i.e., based on sequencing only the C-terminal region of the B646L gene) classification approach, even when updated, still provides only a narrow view of the pathogen’s genome diversity. Therefore, reliance on p72-based genotyping alone is insufficient, highlighting the need for whole genome-based classification methods.

Whole genome sequencing (WGS) and classification by contrast allow for a comprehensive analysis of genomic variation within and between ASFV genomes, offering a broader insight into viral diversity, evolutionary mechanisms, and phylogenetic relationships^21^. Importantly, WGS better empowers the detection of novel or diverged variants, including those due to large insertions, deletions, recombination, and other structural variations^22–24^, which may elude the gene-based classification method^20^. A practical application of this is the whole genome-based biotyping (a term the research group used to distinguish this method of ASFV classification from the classical p72-based genotyping) method mentioned earlier. This method is a whole-genome-based proteome-level characterization of the pathogen^19^. The method was able to detect six biotypes similar in number to the six genotypes described by the updated p72 classification, even though the genotypes don’t completely overlap with the biotypes^19,20^. Furthermore, it was also able to detect and classify variants that are recombinants between biotypes I and II (an insight that was missed by the updated p72-based genotyping, as it grouped these variants into purely genotype II), bringing the total number of ASFV biotypes to seven. Hence, demonstrating the robust nature of whole-genome-based classification methods.

In the regional context, ASF was first introduced into West Africa around the late 1950s, with early outbreaks reported in countries such as Senegal and later Cape Verde, Gambia, and Guinea- Bissau^25–27^. A second and more widely spread outbreak occurred across the region in the 1990s^27–29^. So, although there has been a long-established presence and diversity of the pathogen in the East and parts of Southern Africa, where the ancient sylvatic cycle maintains it, in West Africa, the pathogen is less diverse and relatively recently introduced^30^. ASFV in West Africa has been largely dominated by p72 genotype I, which is considered endemic in some countries within the region^29,31^. However, recent reports of genotype II in countries such as Nigeria, Burkina Faso, Côte d’Ivoire, and other West African countries suggest an evolving genotype dynamics within the region^32,33^.

In Nigeria specifically, despite over 20 years of devastating socio-economic and food security impacts from recurrent ASF outbreaks, only a handful of molecular studies, mainly using gene sequencing, have been conducted^32,34–36^. These have identified both p72 genotype I^34–36^ and II^32^. On the whole genome level, only a few ASFV whole genome-based characterization studies are currently available from Nigeria^37,38^, indicative of an underrepresentation of genomic insights from this region. To address this critical gap, we generated whole-genome sequences from ASFV isolates obtained during recent outbreaks in Nigeria. This effort contributes to the much-needed high-quality genomic data essential for vaccine, diagnostic, and other countermeasure development. This data also forms a part of the foundational datasets required for further comparative and functional analyses. We then proceeded to characterize the genotype, biotype, evolutionary mechanisms, and genomic diversity of the outbreak-causing samples. We also compared these samples with previously published ASFV genomes globally to understand phylogenetic relationships, infer relatedness, and inform the impact of these on countermeasures. Collectively, the findings from this study expand the understanding of ASFV genomics in Nigeria, while also contributing important data and insights to the understanding of ASFV evolution, diversity, and control globally.

## Materials and methods

### Sample selection for sequencing

**Figure 1:**
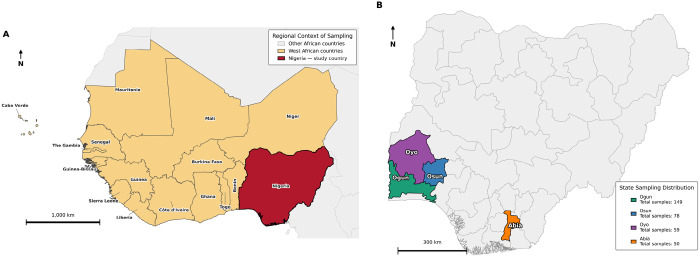
Geographic context and state-level distribution of ASFV samples in Nigeria. **(A)** Map of West Africa showing all countries in the region, with Nigeria highlighted as the study country. **(B)** Map of Nigeria showing the four states where sampling was conducted: Ogun, Osun, Oyo, and Abia. The legend summarizes the total number of samples collected from each state.

From 2021 to 2023, both suspected outbreaks (July-September 2021, September 2022, and July-August 2023) and non-outbreak periods (January–July 2023) of ASF were investigated across four Southern Nigerian states (Fig. 1). During these periods, a total of 336 samples were collected from 54 pig farms and 2 abattoirs. From these, a total of 89 samples were selected from the outbreak periods only: 79 blood and 10 spleen samples **(Table S1)**. This selection was informed by a previous study, where we observed that all non-outbreak samples were negative for ASFV by polymerase chain reaction and IgG ELISA^39^. ASFV genomes generated from the selected samples were subsequently used in this study for genomic characterization of the outbreak-causing pathogen.

- Map of West Africa showing all countries in the region, with Nigeria highlighted as the study country.
- Map of Nigeria showing the four states where sampling was conducted: Ogun, Osun, Oyo, and Abia. The legend summarizes the total number of samples collected from each state.

#### Sequencing protocols

Two sequencing platforms were employed: Oxford Nanopore Technologies (ONT) and Illumina. A total of 50 samples were sequenced using the ONT platform, of which 11 of these overlapped with those sequenced using the Illumina platform. Similarly, 50 samples were sequenced using the Illumina platform **(Table S1)**, bringing the total number of sequenced samples across both platforms to eighty-nine.

#### DNA extraction, enrichment, library preparation, and sequencing

For ONT sequencing, DNA was extracted using the QIAamp DNA Mini Kit (Qiagen; Hilden, Germany) according to the manufacturer’s protocol. DNA quantity was measured using the Qubit dsDNA High Sensitivity Assay Kit (Invitrogen; Waltham, MA, USA). Viral pathogen DNA enrichment employed the NEBNext Microbiome DNA Enrichment Kit (New England BioLabs; Ipswich, MA, USA) to enrich unmethylated DNA while minimizing host and other methylated DNA contamination^40^.

A whole-genome shotgun (WGSG) library preparation method was employed for ONT sequencing, where the enriched DNA was enzymatically fragmented, barcoded using the Rapid PCR Barcoding Kit (SQK-RPB004), and then ligated with rapid sequencing adapters. The prepared libraries were subsequently pooled and loaded onto a flow cell for sequencing on the GridION platform (Oxford Nanopore Technologies; Oxford, UK). Raw FASTQ files were generated using MinKNOW and only reads passing default quality filters were retained for downstream analysis.

For Illumina sequencing, DNA was extracted using the MagMax Core RNA/DNA Kit on a MagMax Express magnetic bead robotic platform (Applied Biosystems, Thermo Fisher Scientific, CA, USA). DNA quantity was measured using the same Qubit dsDNA High Sensitivity Assay Kit. Illumina library preparation followed two approaches: (i) Tiled Amplification (TA): Extracted DNA was initially enriched^41^. The resulting products were then purified with Ampure XP beads, quantified, and used to construct libraries with the Nextera XT Kit (Illumina; San Diego, CA, USA). (ii) Whole Genome Shotgun (WGSG): Extracted DNA was directly processed with the Nextera XT Kit without prior amplification. Sequencing was performed using NextSeq 2000 P3 reagents (300 cycles) on an Illumina NextSeq 2000 platform.

#### Bioinformatic analysis

##### Genome assembly, biotype assignment, and variant characterization

For the ONT workflow, reads from each sample were aligned to ASFV RefSeq genomes using Minimap2 v2.28^42^. The reference with the highest genome coverage and percentage identity was selected for genotype assignment^39^. Variants, including SNPs and insertions/deletions (InDels), were detected with either Freebayes v1.3.6 (-p 1 --standard-filters --min-coverage 6 or Ivar v1.4.3 (-m 100 -q 8 -t 0.6)^43,44^, and a custom open-source tool (https://github.com/lakinsm/simple-snp). InDels were manually verified, and consensus sequences were generated using either BCFTools (v1.17) or Ivar v1.4.3 (-q 8 -t 0.6 -c 0.8 -m 100)^45^.

Consensus genomes with average depth ≥ 6× or minimum read depth ≥ 100× with < 10% ambiguous bases (N) were retained for further downstream analysis. Those with > 10% but ≤ 40% Ns were considered partial and unfit for downstream analysis. To further verify the genotype assigned to our genomes during the assembly stage, and to rule out the presence of recombination, a biotype assignment tool (designed based on comparing and grouping ASFV proteomes through unsupervised machine learning) was used to characterize our genomes into biotypes^19^.

For the Illumina workflow, the same reference-guided assembly approach was employed to generate high-quality ASFV genomes. Illumina reads from each sample were aligned to ASFV reference genomes from GenBank using Minimap2 v2.30. Variants were called using iVar v1.4.3 (-m 20 -q 30 -t 0.6), and consensus sequences were generated (-q 30 -t 0.6 -c 0.8 -m 20). Genotype and biotype assignment were performed just as for the ONT sequences. Genomes containing < 10% ambiguous bases (N) were considered high-quality and full-length, whereas those with > 10% but ≤ 40% Ns were categorized as partial and excluded from downstream analyses. Variant annotation was performed using SnpEff v5.2f^46^. In-house scripts were used to summarize and visualize annotations both within and across samples.

##### Phylogenomic analysis

High-quality consensus sequences generated from both ONT and Illumina workflows were subjected to comparative phylogenomic analysis to assess their evolutionary relationships with publicly available ASFV genomes belonging to the same biotype as ours (all existing ASFV datasets as of 15th January, 2026, were downloaded from GenBank. These were then biotyped, and only those belonging to biotype II were extracted for downstream analysis). Multiple sequence alignment was performed using MAFFT^47^, followed by manual curation to remove poorly aligned regions or sequences introducing misalignments. Maximum likelihood phylogenetic trees were reconstructed using IQ-TREE, with automatic model selection and 1000 ultrafast bootstrap resampling to assess branch support^48,49^. Phylogenetic trees were visualized using iTOL v7.2.2^50^.

#### Ethics statement

The study was conducted in accordance with national and institutional guidelines, with ethical approval obtained from the National Health Research Ethics Committee (NHREC) (NHREC/01/01/2007) and the Animal Care and Use Committee (AUCC) (AEC/02/123/22) in Nigeria. All sampling procedures adhered to animal welfare standards and were performed exclusively by licensed veterinarians. Before sample collection, farmers and pig owners were informed of the survey’s objectives and rationale. Participation was voluntary, and individuals were free to withdraw at any point.

## Results

### Genome assembly and classification

For the ONT workflow, of the 50 ONT-sequenced samples, 23 (46%) yielded full-length genomes **(Table S1)**. All aligned best to the Georgia 2007/1 reference (NC_044959.2) and were thus classified as genotype II. Biotype classification also revealed that all our samples belonged to biotype II without any known recombination. Their genome lengths range from 176,705 to 184,104 bp (excluding ambiguous bases), and with genome coverages of 95.5–96.6%.

For the Illumina workflow, however, reference-guided assemblies produced 20 genomes (40% of sequenced samples), all mapping best to the ASFV Georgia 2007 reference (NC_044959.2) and also classified as genotype II. Biotype assignment also revealed that all the samples belonged to biotype II without any known recombination. This workflow produced 7 full and 13 partial genomes **(Table S1)**. The full genomes had lengths ranging from 175,160 to 182,829 bp (excluding ambiguous bases), corresponding to genome coverages of approximately 91.9–95.9%.

Notably, three of the 23 genomes generated from the ONT workflow overlapped with those from Illumina, bringing the total number of full genomes generated to 27 **(Table S1)**.

### Single Nucleotide Polymorphisms (SNPs), insertions, and deletions

For the ONT workflow, more than 200 polymorphic sites were detected across coding and non-coding regions. The most affected genes included *MGF_360–2L, MGF_360–21R*, and *ASFV_G_ACD_00120*. Additional polymorphisms were detected in *EP402R, MGF_110–4L, MGF_360–8L, B602L*, and *D345* genes. Details are provided in Supplementary Files **S1 and S2**.

In addition, seventeen of the 23 ONT assemblies revealed large deletions in three genomic regions:

- Left Variable Region-1/LVR-1 (8566–9197 bp): Occurred in 17.6% of the samples and was observed exclusively in samples from Osun State.
- Left Variable Region-2/LVR-2 (11761–18295 bp): Occurred in 100% of the samples and was observed in samples from both Ogun and Osun states.
- Right Variable Region/RVR (188922–190584 bp): Occurred in 70.6% of the samples and was also observed in both Ogun and Osun State samples.

As for the Illumina workflow, between 66 and 99 SNPs were identified across the full genomes (i.e., within annotated coding regions/genes), with at least 33 consistently present in all assembled genomes. Most SNPs, irrespective of sampling location, occurred within multigene family genes (*MGF_360–21R, MGF_360–2L*, and *MGF_110–4L*), but polymorphisms were also detected in well-studied functionally annotated genes such as *EP402R* (CD2v protein) and *KP177R* (p22 protein), where they produced missense substitutions. Also, across all study genomes, at least 20 genes had SNPs resulting in missense substitutions. Small indels (< 50 bp), with some causing frameshifts, were also observed. Ten genes across all samples were observed to have insertions relative to the reference, while only one gene was observed to have a deletion across all samples (i.e., *MGF_300–2R*). Detailed SNP and small indel data, including affected gene, their distribution across samples, and inferred functional impacts, are provided in Supplementary Files **S3 and S4**.

### Phylogenomic analysis

**Figure 2:**
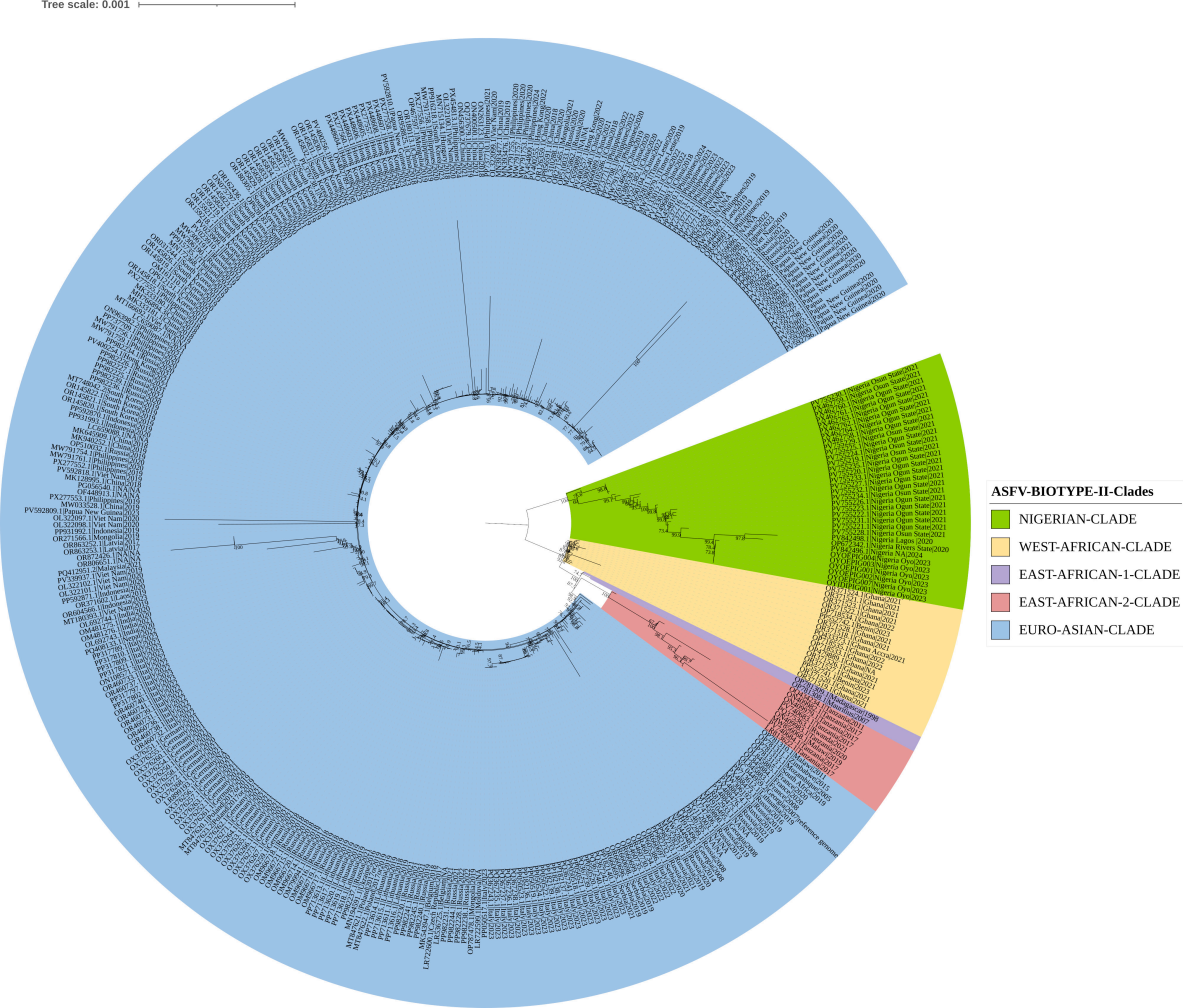
Maximum likelihood phylogeny of Biotype II ASFV genomes. The Nigerian clade is diverged significantly from all other clades but is most related to the West African Clade as indicated by the long internal branch (substitutions per site) separating the Nigerian Clade from all other clades. This mid-point rooted (for visualization) tree also shows a clear geographic clustering amongst biotype II sequences, with clades colored according to their spatial clustering patterns. Bootstrap support values (>60) are visualized at internal nodes with separate colors used to delineate clades.

Phylogenomic analysis of ASFV genomes revealed the distinct clustering of Nigerian genomes within the biotype II sequences. When compared with the publicly available ASFV genomes, our study genomes and those from other Nigerian studies diverged into a distinct clade relative to all other biotype II genomes from the NCBI GenBank database as depicted by a well-supported node bootstrap value (100%) and a long internal branch (Fig. 2). This is further contextualized by the finding that, although the Nigerian genomes are most closely related to those from other West-African countries such as Ghana and Benin Republic, they diverge considerably from these genomes (by approximately 113 SNPs).

Within the Nigerian clade, we also observed some diversity and clustering patterns; firstly, the relatedness between the common ancestor of these internal clade structures and the common ancestor of the entire Nigerian clade is poorly supported (60%). Hence, characterizing the observed clustering patterns within the Nigerian Clade as well-defined subclades is not advisable. However, within this clustering pattern, we observed that Osun and Ogun samples are largely closely related to each other, with only a few instances of significant internal branching (constituting approximately 19–133 SNP differences between the clusters). However, despite this divergence, there are no clear spatial clustering differences observed between the genomes from both states, thus indicating a common transmission event or source. The same was not observed for sequences from Rivers, Lagos, and Oyo States, as genomes from these states considerably diverged from each other, with clear differences in spatial clustering when compared to Osun and Ogun samples.

Despite using the Georgia 2007/1 reference genome for our assembly, we observed a substantial divergence from this reference, with the reference clustering within the Euro-Asia Clade. In addition, a clear geographic clustering pattern was evident across the phylogeny, with Nigerian, other West African, East African, and Euro-Asian isolates forming separate, well supported clades.

## Discussion

This study presents the first in-country sequenced and analyzed ASFV genomes from Nigeria and constitutes the largest ASFV genomic dataset generated to date in both Nigeria and Africa. By assembling 23 ONT and 7 Illumina full genomes, we begin to address the critical gap in ASFV genomic data from West Africa - a region historically underrepresented in global ASFV genomics. We employed a whole-genome sequencing approach to genotyping and biotyping the outbreak-causing strain, while also simultaneously providing an extensive report of the associated mutations (SNPs and indels) and their potential functional implications. While functional validation of these mutations will be required, our findings offer valuable preliminary insights into ASFV evolution in Nigeria. We confirmed that the biotype/genotype II sequences associated with the 2020 through 2024 outbreaks in southwestern Nigeria all cluster within the same distinct clade (suggesting a similar and/or likely persistent transmission) but are also divergent from each other and from the global biotype II genomes. Our findings suggest that this divergence may influence vaccine design, viral serotyping efforts, diagnostics, and other translational endeavors.

Both Illumina and ONT assemblies confirmed ASFV genotype and biotype II as the pathogen type circulating in Nigeria, consistent with earlier reports^39,37,32^. Importantly, these variants show no evidence of known recombination patterns between genotype I and II. This suggests that clinical manifestations and countermeasure designs would not be significantly impacted by this evolutionary pattern. Notably, ~ 4–8% of the Georgia 2007 reference could not be mapped by our sequencing reads, reflecting novel large deletions in Nigerian genomes relative to the reference. We identified deletions in LVR-1, LVR-2, and RVR, extending beyond previously reported deletions^37^ in Nigeria. The LVR-2 deletion (~ 14 genes affected) includes genes such as *MGF_110–10L* and *MGF_110–14L*, previously linked to reduced virulence in Estonian strains^24^. However, Ambagala *et al*., (2023) demonstrated that despite similar deletions, Nigerian strains retained their virulence upon pig inoculation^37^. The LVR-1 deletion (~ 631 bp) involved *MGF_110–3L*, *ASFV_G_ACD_00120*, and *MGF_110–4L* genes. Similar deletions have also been reported in previous studies^51–53^. However, whether this produced an in-frame fusion in our study samples, just as described by Ambagala *et al*., (2023)^37^ remains unclear. The RVR deletion (reported here for the first time in Nigeria) affected four genes, including *MGF_360–21R*, for which a prior study suggests its deletion may play a role in ASFV adaptation in wild boar^54^. Observing the presence of this mutation in 70.6% of ONT sequences used to detect large deletions may indicate the role of the wild boar-habitat transmission cycle in Nigerian ASFV outbreaks. This may be because the variants causing this outbreak were imported from regions where wild boar played a role in the disease transmission. Collectively, these deletions may therefore influence pathogen virulence, host adaptation, or even the performance of genomic sequencing resources such as primers or probes designed for amplicon-based or hybrid-capture sequencing methods.

SNPs identified in our study genomes, although mainly clustered in multigene families (*MGF_360, MGF_110, MGF_505*), were also found in functionally critical genes such as *EP402R* (CD2v) and *KP177R* (p22). In the latter genes, variants at genomic coordinates NC_044959.2:75251:T > G, NC_044959.2:75291:C > T, and NC_044959.2:4484:A > G, respectively, resulted in missense mutations. Given *EP402R*’s central role in serotyping^55,56^, *KP177R* projected roles in serological surveys and future vaccine strain differentiation^57,58^, and the involvement of some multigene family genes in tick replication^59^, macrophage infection^60,61^, and ASFV virulence^62,63^, these mutations are likely to impact viral characterization efforts, surveillance, and phenotype, respectively. These findings, together with the large number of SNP changes reported in this study **(Supplementary Files S1–S4)**, further raises concerns about the performance of detection and other control tools. Even more concerning is the potential for cross-host spillover, since many of the virus entry pathways and receptors utilized by ASFV in pigs are also present in other eukaryotic organisms. Large-scale mutations across the pathogen’s genome may therefore result in non-suid adapted strains. This warrants continuous ASFV genomic surveillance in non-suid hosts, particularly those in close interaction with suids, to generate real-time and robust information that keeps us a step ahead of the pathogen.

ASFV sequences from this study, despite being collected from geographically distinct locations and at different outbreak periods, still fall within the same clade as genomes sequenced from Nigeria previously, indicating a shared transmission and/or possible local persistence. Geographically, this is especially inferred from the clustering pattern observed between genomes from Osun and Ogun States. Although collected from different locations (within the same year, 2021), these cluster without any clear spatial differences. This picture suggests that Osun and Ogun States most probably had a shared transmission event. ASFV genomes from states like Lagos, Oyo, and Rivers, on the other hand, show spatial clustering (separated from the Ogun and Osun samples by long internal branches—especially Rivers and Lagos genomes). However, they are still within the larger Nigerian clade, thus supporting our local persistence theory. Diverged significantly from others, the Rivers and Lagos sequences could represent either rapid within-host or even between-host evolution. This is because ASFV is a rapidly transmitted virus with the capacity to also infect large number of animals (up to 90% of the susceptible population) at a given time. Thus, within a short period of time, an introduced variant (from a single source) can end up diverging significantly from its original form, leading to the circulation of distinct ASFV variants within the region. Additionally, unlike what is generally expected that DNA viruses should evolve slowly compared to RNA viruses, ASFV has been found to have a very unstable genome with some sites within the genome evolving at rates similar to RNA viruses^64,65^. These reasons could therefore explain the variation observed amongst the genomes within the Nigerian Clade, given the short time interval between them. That being said, the limited representation of these genomes (i.e., from Lagos and Rivers) stifles our capacity to reach convincing inferences, as more samples from these locations may tell a different relationship story. Notwithstanding, the current relatedness and relative divergence between samples from both states and those from Ogun, Osun, and Oyo states are well supported (bootstrap > 80%). It is important to note, however, that the direction of viral spread (i.e., from one Nigerian state to another or vice versa) could not be determined due to the limitations of the maximum likelihood models used in this study.

In relation to regional (African) biotype II sequences, the Nigerian genomes in this study show closer relatedness to sequences from neighboring West African countries. This observation improves on a previous suggestion by Ambagala *et al*., (2023), which proposed a potential East or Southern African ancestral source for the Nigerian sequences^37^. In the present study, we do not attempt to infer ancestral origins (due to the limitation of maximum likelihood trees), but rather focus on describing genetic relatedness and relative distances between clades. Our analyses thus indicates that Nigerian ASFV genomes are more closely related to the West African and Euro-Asian genomes than to those from East Africa. The inclusion of the Nigerian sequence reported by Ambagala *et al*., (2023) (accession OP672342.1) within our dataset also supports this relatedness. This pattern appears to be consistent with previous regional transmission patterns linking ASFV spread in Nigeria to bordering West African nations^10^. However, given that sequences from the neighboring West African countries were collected within a similar timeframe (i.e., 2020–2024) as the Nigerian samples and yet do not cluster with the Nigerian sequences this suggests the possibility of distinct transmission events originating from a shared but currently unidentified source. The observed genetic distances between the clades (i.e., Nigerian and larger West African) suggest divergence that may reflect differing ecological or epidemiological pressures across locations. Notably, the West African clade, comprising primarily sequences from Ghana and the Benin Republic, forms a relatively tight cluster with limited divergence, supporting the possibility of localized and unique transmission within this group compared to the Nigerian clade.

Finally, with respect to the global perspective, distinct spatial clustering patterns were observed with each region’s sequences clustering differently from one another. The Nigerian, other West-African, East African (1 and 2), and the Euro Asian genomes all cluster separately. The only exception to this rule is Southern-African samples, which were observed to cluster with the Euro-Asian clade. This observation was, however, not unexpected, as this set of sequences had been implicated by a previous study to have been the source of ASFV spread from Africa to the European continent^66^. We hypothesize that the trade restrictions placed on countries once they are denoted ASFV positive may be one of the factors responsible for this geographically inclined divergence. As pigs become geographically constrained, leading to ASFV from each locality being placed under separate host and ecological factors, it is also expected that viruses would evolve differently. More importantly, the distinct geographic clustering observed in this study indicates that vaccines, diagnostics, and other countermeasures must account for ASFV regional and local divergence for optimal effectiveness.

This is particularly relevant for vaccine design. The most recent ASFV vaccines approved for use in Vietnam were developed from the Georgia strain’s backbone (ASFV-G), the predominant variant across the Euro-Asian region^67,68^. This same genome was used as the reference in our assembly and phylogenetic analyses. However, both variant characterization and phylogenetic analyses revealed that our study sequences and the entire Nigerian Clade are diverged considerably from this genome. In designing the vaccines, deletions of specific genes from this strain conferred protection to pigs^67,68^. However, since our analyses confirmed a clear divergence between this reference and strains circulating in West Africa, it follows that vaccine development efforts in our region should ideally be based on locally circulating strains. While the same principle of gene deletions coupled with rigorous testing and validation may be applicable, using indigenous isolates as templates would likely yield more effective and regionally adapted vaccines for our pig populations. In conclusion, this study presents the largest ASFV genomic dataset from Nigeria and Africa to date, providing key insights into the genotype, biotype, mutations, and genetic diversity of outbreak-causing strains within our study region. By characterizing mutations and clade structures, it underscores the importance of genomic surveillance in expanding epidemiological insight, guiding vaccine development, diagnostics, and other countermeasure strategies. Overall, our findings lay a foundation for future research and support efforts that would ultimately lead to the protection of both the local and the global swine industry.

## Supplementary Material

Supplementary Files

This is a list of supplementary files associated with this preprint. Click to download.

- SupplementaryInformationfiles.zip

## Data Availability

The datasets generated during this study have been deposited in GenBank and are available under accession numbers (PV755219-PV755235 and PX462758-PX462764). Additional ASFV genomic sequences were obtained from publicly available GenBank resources and are cited accordingly in the manuscript. All other data supporting the findings of this study are included within the article and its supplementary materials.
